# Effects of language background on executive function: Transfer across task and modality

**DOI:** 10.3389/fpsyg.2022.923123

**Published:** 2023-01-05

**Authors:** Yeonwoo Kim, Zixuan Ye, Zachary Leventhal, Wei-Ju Wang, Erik D. Thiessen

**Affiliations:** ^1^Department of Psychology, Carnegie Mellon University, Pittsburgh, PA, United States; ^2^Department of Statistics and Data Science, Carnegie Mellon University, Pittsburgh, PA, United States; ^3^Department of Economics, Carnegie Mellon University, Pittsburgh, PA, United States

**Keywords:** cognition, bilingual, executive function, switch costs, modality

## Abstract

The relation between linguistic experience and cognitive function has been of great interest, but recent investigations of this question have produced widely disparate results, ranging from proposals for a “bilingual advantage,” to a “bilingual disadvantage,” to claims of no difference at all as a function of language. There are many possible sources for this lack of consensus, including the heterogeneity of bilingual populations, and the choice of different tasks and implementations across labs. We propose that another reason for this inconsistency is the task demands of transferring from linguistic experience to laboratory tasks can differ greatly as the task is modified. In this study, we show that task modality (visual, audio, and orthographic) can yield different patterns of performance between monolingual and multilingual participants. The very same task can show similarities or differences in performance, as a function of modality. In turn, this may be explained by the distance of transfer – how close (or far) the laboratory task is to the day to day lived experience of language usage. We suggest that embodiment may provide a useful framework for thinking about task transfer by helping to define the processes of linguistic production and comprehension in ways that are easily connected to task manipulations.

## Introduction

Perhaps due to the close interrelation between language and thought (e.g., Chomsky, 1964), researchers have long suspected a link between linguistic experience and cognitive abilities. However, the nature of this hypothesized link has varied widely across time and contexts. Early in the 20^th^ century, researchers and educators in the United States confidently pronounced that monolingualism was the “correct” way to raise children, and any deviations from this standard increased the risk of developmental delays or disorders (e.g., Saer, 1923; Yoshioka, 1929). Within decades, the opinion among researchers changed dramatically, due to pioneering research by Peal and Lambert (1962), a tradition subsequently much expanded by Bialystok (2017) and Bialystok et al. (2007) which provided evidence that bilingualism actually promotes beneficial cognitive outcomes in executive function skills, metalinguistic awareness, and cognitive flexibility, as well as evidence that bilingualism protects against cognitive decline and dementia (for review, see Adesope et al., 2010). In particular, the *cognitive reserve* framework has provided a conceptual account for understanding the protective effects of bilingualism, especially in aging (see Bialystok, 2021). This framework suggests that the protective effects of bilingualism result from the lifelong experience of resolving competition between the two (or more) jointly activated languages with which bilinguals are familiar. This results in a series of adaptations to cognitive and neurological systems creating conditions in which cognitive actions are more automatic and less effortful (Cabeza et al., 2018).

While much research has supported the cognitive reserve framework, or related bilingual cognitive advantages, there have been notable exceptions. Objections to the perspective have been made from both theoretical and empirical grounds. Bialystok (2017) have argued that the lifelong experience of selection between languages provides multilingual speakers with some generalized advantage in attention, inhibition, or selection. From this perspective, language selection is explicitly embedded within a domain-general process of executive function (e.g., Blumenfeld and Marian, 2013; Paap et al., 2018). However, the idea of a unified set of executive function processes is itself a complex and disputed claim. For example, there are few (if any) process-pure measures of executive function abilities; instead, tasks are likely to involve multiple executive function skills in different degrees (for discussion, see Miyake et al., 2000). This task impurity problem makes it difficult to generate *a priori* predictions about which tasks should, or should not, show effects of multilingual experience (for discussion, see Hartsuiker, 2015).

A related objection to the cognitive reserve framework is that the phenomenon itself is not robust or replicable. A number of investigators have reported failures to replicate the phenomenon of a multilingual advantage (e.g., Paap and Greenberg, 2013; Gathercole et al., 2014). These non-replications raise the possibility that any bilingual advantage is too small or unreliable to be of practical significance, or perhaps even an illusion. It should be noted that many of these non-replications of a bilingual advantage (though not all; e.g., Antón et al., 2016) have been obtained with undergraduate or healthy adult participants. The cognitive reserve framework explicitly argues that the “protective” or “beneficial” effects of bilingualism should be most apparent in situations where cognitive resources are heavily taxed or drained. Therefore, the framework predicts that healthy young adults are the group least likely to show the beneficial or protective effects of bilingual experience, which are thought to be more pronounced in children and older adults (e.g., Craik et al., 2010; Bialystok, 2021). Consistent with this, a recent meta-analysis (Ware et al., 2020) indicated that evidence for a bilingual advantage is stronger for participants over 50 than for participants between the ages of 18 and 29.

At minimum, then, it is apparent the bilingual advantage is not always easily detected, or is more easily observed in some contexts than others. As such, making *a priori* predictions about when one should (or should not) expect to find differences as a function of language background is of premium importance in theory testing. We propose that an important factor to consider in making these predictions is the “distance of transfer.” Transfer refers to the process of executing some learned behavior or process in a novel context; for example, after learning to golf on one course, playing a new course requires transferring those learned golf skills to a new setting (for discussion, see Salomon and Perkins, 1989). In some cases, transfer of learning only requires adapting to superficial, perceptual differences in context or setting, which is referred to as “near” transfer. In other cases, learners might be asked to apply their skills in settings that are quite distinct from their training. For example, after being trained on a working memory task with numerical digits, being asked to perform a working memory task with orthographic numbers would be a case of near transfer, while being asked to remember chess displays would be a case of far transfer. Training in executive function skills—of the type invoked by the cognitive reserve framework and its critics—typically results in near transfer, rather than far transfer (Kassai et al., 2019; Sala and Gobet, 2019; Gobet and Sala, 2020). Evidence for far transfer is rare, a finding that appears to hold across the lifespan (Sala et al., 2019).

Consideration of transfer suggests that if there is an effect of bilingualism on executive function, we are much more likely to observe it in tasks or settings that are very similar to (multi-) language use, and less likely to observe it in contexts that are less similar to linguistic stimuli, tasks, or processes. Of course, this requires us to define some framework or property structure along which to evaluate similarity; in isolation, the notion of similarity is notoriously susceptible to circularity (Goodman, 1972; Tversky, 1977). To ground our perspective on similarity, we will rely on the embodiment theory of cognition, which argues that the mind’s experience of cognition is deeply rooted in the body’s interactions with the world (e.g., Barsalou, 1999a). That is, when comprehending or producing a verb like “open,” humans do not rely on an abstract, symbolic, or propositional definition of the verb; instead, they recall their physical experiences with opening doors, drawers, or containers (Barsalou, 1999b). In this way, cognition is always contextually situated, and the critical factors that define similarity are the (embodied) representations invoked in a task, and the operations over those representations. This is in some ways antithetical to the claims of the domain-general executive function “reserve” invoked by the cognitive reserve framework (Bialystok, 2017). However, we believe it may be a better fit to the (somewhat contradictory) state of the literature, in part by providing an opportunity to generate explanations and predictions about the replications and non-replications of bilingual advantage in executive function.

From the embodiment perspective, the inconsistent pattern in the literature—with both replications and non-replications of a bilingual advantage—may be related not only to differences in the participant population (young adults, as opposed to children or older adults), but also due to differences in the distance of transfer between the experience of bilingualism and the laboratory task(s) used by specific laboratories or investigators. The current investigation was conducted to investigate the plausibility of this claim. In particular, we examined the prediction that the more “language-like” a task is, the more likely it is to show evidence of a difference in performance between monolingual and multilingual speakers. That is, the more language-like a task is, the more likely it should be to provide replicable evidence of some effect of linguistic background.

Our goal in this study is to assess whether evidence of effects of linguistic background on executive processing are more or less observable as a function of the linguistic nature of the task and stimuli. Because executive function is a broad umbrella concept, we particularly focus our attention on the construct of “response selection,” which has often been described as a key shared component between multilingual experience and executive function tasks. The cognitive reserve framework argues that it is the lifelong experience of regularly being asked to disengage attention from the non-target information (i.e., language), and to switch attention to relevant information, that is the mechanism responsible for subsequent advantages in executive function (Bialystok, 2009). This is specifically an appeal to domain-general attentional processes, rather than to domain-specific (e.g., perceptual) inhibitory response (c.f. Freeman et al., 2017; Paap et al., 2018). As such, we will present participants with two response selection tasks that have been suggested to be related to the attentional control skills accentuated by multilingual experience: the Simon task (Simon and Small, 1969) and the dimension-switching task (Prior and MacWhinney, 2010).

In addition to assessing participants ability to perform conflict resolution in a relatively decontextualized laboratory task, we will also ask participants to perform conflict resolution in a (somewhat) more linguistically-relevant task: word segmentation (e.g., Saffran et al., 1996). The ability to segment words from fluent speech is a fundamental linguistic skill, and one that reflects a listener’s fluency with the language. Because words in fluent speech are not consistently marked by pauses or any other acoustic feature (e.g., Cole and Jakimik, 1980), listeners must rely on probabilistic cues—such as phonological structure—to identify where words begin and end in fluent speech (e.g., Johnson and Jusczyk, 2001). Fluent speakers quickly and automatically integrate information across multiple probabilistic cues to identify the most likely word boundaries in an utterance (e.g., Nazzi et al., 2014). In some cases, because cues are probabilistic, they may indicate different word boundaries. For example, most content words in English are stressed on their first syllable, and as such English speakers treat lexical stress as a cue to word onset (e.g., Jusczyk et al., 1993). Nevertheless, in a word like *giraffe*, where stress falls on the second syllable, listeners must be able to rely on other cues to correctly segment the word.

As this example shows, probabilistic cues can conflict and compete with each other, and successful word segmentation involves resolving this competition. Prior research has found that the ability to perform conflict resolution in laboratory tasks is predictive of the ability to perform conflict resolution in the word segmentation task, and successfully learn words (Weiss et al., 2010). This relationship has been observed for both monolingual and multilingual speakers (Bartolotti et al., 2011). We will attempt to replicate this relationship in the current investigation, in particular following the Weiss et al. (2010) methodology. The fact that this replication involves a task—word segmentation—that is fundamentally grounded in real language comprehension and processing should make this an instance of relatively near transfer, and thus perhaps more replicable than other tasks.

Finally, as a direct test of the hypothesis that transfer distance matters, we will explicitly manipulate the linguistic nature of the stimuli used in the dimension-switching task. The majority of research investigating the effects of linguistic experience on conflict resolution has relied upon tasks that involve visual stimuli. Relatively less work has examined performance with auditory tasks, though some systematic investigations of stimulus and task modality have been attempted (e.g., Calabria et al., 2012; Foy and Mann, 2014; MacNamara and Conway, 2014). We hypothesize that language background will more strongly influence participants’ performance in a task with linguistic stimuli (words) than in a task with non-linguistic stimuli (images). This hypothesis is based on prior evidence that training of executive function abilities largely results in close transfer (that is, improvements on tasks that are very similar to the training experience), and only more rarely gives rise to far transfer (e.g., Kassai et al., 2019). If experience with multiple languages does indeed train executive function, that training should have greater efficacy for tasks that are similar to the training—that is, tasks that involve linguistic stimuli or linguistic processes. Tasks that involve non-linguistic stimuli or processes should show less effect of prior linguistic background. To test this hypothesis, we will present participants with a dimension-switching task, one that has previously been used to investigate differences between monolingual and multilingual participants (e.g., Prior and MacWhinney, 2010). In this task, participants must switch between rating stimuli on one dimension (living/non-living) to rating stimuli on another dimension (large/small). Consistent with prior work, we expect that monolingual participants will show a larger switch cost (i.e., slower responses) when prompted to switch from one rating dimension to the other, while multilingual participants should show a smaller switch cost. Of particular interest to us is whether the advantage in switch cost for multilingual participants (compared to monolingual participants) differs as a function of whether the rated stimuli are words or images.

To investigate these questions, we recruited a large sample of undergraduate participants, and asked them to provide us with extensive information about their previous language usage. With this information, we sorted participants into categories (monolingual, bilingual, trilingual, etc.), as well as analyzed individual differences in language background as a continuous variable. All participants completed a battery of tests assessing working memory, for which we do not expect to find differences as a function of language background (e.g., Lehtonen et al., 2018; Antón et al., 2019); this battery provides some measure of information about whether our participant groups are well matched on dimensions other than language background. All of these participants then completed the Simon task, and a word segmentation task with either converging or conflicting cues (e.g., Weiss et al., 2010). Finally, participants completed the task-switching task with either word or image stimuli. Taken together, these tasks will provide us with insight on the replicability and domain generality of effects of linguistic background on executive function skills.

## Materials and methods

### Participants

Two hundred and eighteen introductory psychology course students enrolled at Carnegie Mellon University, aged 18–25 years, participated for class credit (141 female, 70 male, 5 nonbinary, 2 declined to disclose). We excluded 10 participants who did not complete all the tasks from the main analyses, resulting in 208 participants in our final sample. Of these 208 participants, 108 were of East Asian origin, 64 were White, 22 were South Asian, 18 were of Hispanic, Latino, or Spanish origin, 10 were Black or African American, 8 were Southeast Asian, and 2 were Middle Eastern or North African (Note: participants were allowed to select multiple races, resulting in a sum greater than 208). Fifty participants reported that they were monolingual English speakers. Of the remaining multilingual participants, 95 identified as bilingual, 50 identified as trilingual, and 13 participants rated themselves as familiar with four or more languages. Descriptive statistics on the age of acquisition, usage, and proficiency of each language reported are summarized in Table 1.

**Table 1:** Descriptive statistics of language background (age of acquisition, usage, proficiency) for each language reported.

	LDom1 (*N* = 173)		LDom2 (*N* = 78)		LDom3 (*N* = 24)		LDom4 (*N* = 5)	
Age acquired	1.53	(1.69)	4.27	(3.84)	10.71	(3.97)	11.40	(5.08)
Age Fluent	5.06	(2.43)	9.91	(5.78)	14.04	(4.30)	14.00	(4.30)
Age Reading	4.39	(1.55)	7.50	(4.24)	11.71	(3.45)	11.40	(5.08)
Age Fluent Reading	6.79	(2.32)	11.29	(4.94)	13.46	(4.42)	14.20	(3.35)
Time in Country	17.87	(3.27)	4.96	(6.10)	2.53	(6.36)	4.00	(7.87)
Time with Family	17.00	(5.47)	8.34	(9.22)	1.66	(5.50)	4.40	(7.80)
Time in School Work	15.43	(3.82)	4.62	(4.21)	3.39	(5.44)	4.80	(7.82)
Reading Proficiency	9.63	(0.81)	7.15	(2.35)	6.29	(2.65)	5.40	(2.30)
Speaking Proficiency	9.76	(0.55)	7.08	(1.93)	5.42	(2.26)	4.00	(1.58)
Understanding Proficiency	9.73	(0.63)	8.18	(1.63)	6.33	(2.30)	5.00	(1.73)
TF score	12.97	(0.25)	10.94	(2.43)	8.92	(2.62)	5.80	(2.78)

These data are reported for those participants from whom we have complete data. Means are reported first, with standard deviations inside parentheses. Proficiency measures range from 0 to 10. TF score is the number of “True” responses for the 13 true–false questions assessing language familiarity.

Because multilingual participants are necessarily heterogeneous, we supplemented our categorization of participants as “monolingual” or “multilingual” with a more continuous measure. In recent years, both proponents and skeptics of a bilingual advantage have made attempts to treat language experience as more of a continuum, and less of a categorical variable (e.g., Anderson et al., 2018; Paap et al., 2018; Anderson et al., 2020b). This is consistent with the arguments of embodied cognition, which suggest that abstract categorical variables like “bilingual” are less informative than detailed information about the contexts in which a person uses language, and the tasks they perform with that language. Fortuitously, this continuous perspective on language use is also often advantageous in terms of statistical power (e.g., Peña et al., 2016). By adopting a continuous perspective, we do not mean merely looking at the age at which acquisition of L1 and L2 occurred, or the number of years a participant has spent using their language(s). Rather, our embodiment perspective suggests we should focus on the tasks that language is used with and for. This is in many ways consistent with the “adaptive control hypothesis” (Green and Abutalebi, 2013), which predicts that the neural underpinnings of language control and processing should adapt to the control demands presented by the interaction between multiple known languages (for review, see Abutalebi and Green, 2016). In particular, we will focus on participants’ experience reading (and writing), comprehending, and speaking languages. Information about participants’ language use in these three task domains, as well as the context in which they use language, and the age and amount of use for each language, was collected via a previously validated survey, the Language Experiences and Proficiency Questionnaire (LEAP-Q; Marian et al., 2007).

### Stimuli

#### Word segmentation task

This task was designed following Weiss et al. (2010), but with some alterations to the artificial language on account of constraints of our speech synthesis device. Participants listened to an artificial language composed of four bisyllabic words, bugo (/bu.goʊ/), dapu (/dæ.pu/), diti (/di.ti/) and dobi (/doʊ.bi/). To generate this language, consonant-vowel syllables were synthesized in isolation, at a monotone 230 Hz. These syllables were then concatenated into a sequence with no pauses between syllables, ordered such that each bisyllabic word occurred 90 times, and never followed itself. This artificial language has no acoustic cues to word boundaries, but participants can discover words by attending to the likelihood of syllable co-occurrence. Syllables within a word always predict each other; so, for example, when a participant hears “bu,” they will always hear “go” next. At the end of a word, any of the other three words can occur, so the co-occurrence probabilities are much lower at word boundaries (Aslin et al., 1998).

After creating this artificial language, we modified it further to create two versions: a Convergent Cue version, and a Conflicting Cue version. In the Converging Cue version, pauses were added between the words. Thus, a participant might hear “diti (pause) bugo (pause) dobi (pause)....,” with a pause marking the boundary between words, consistent with the statistical information about syllable co-occurrence.

In the Conflicting Cue version, pauses occurred in the middle of each bisyllabic word. Thus, participants might hear “di (pause) tibu (pause) godi (pause)...,” with the pauses interrupting each (statistically defined) word. In this language, the cues to word boundaries conflict. Co-occurrence cues indicate one set of boundaries, while pause cues indicate a different set of boundaries.

Participants were assigned one of five pause duration conditions: 0 ms (at which duration the Converging Cue and Conflicting Cue languages are identical), 10 ms, 15 ms, 25 ms, and 50 ms (at which duration pilot testing indicated that the co-occurrence cue and the pause cue are of roughly equivalent strength). See Table 2 for the number of participants in each condition, divided into monolingual and multilingual participants.

**Table 2:** (A) Number of monolingual participants in each word segmentation task condition. (B) Number of multilingual participants in each word segmentation task condition.

	Pause duration (milliseconds)					
Language condition	0 ms	10 ms	15 ms	25 ms	50 ms	Total
**(A) Monolingual**
Convergent	6	4	2	2	10	24
Conflicting	8	8	3	4	3	26
Total	14	12	5	6	13	50
**(B) Multilingual**						
Convergent	15	7	14	11	29	76
Conflicting	17	15	17	17	16	82
Total	32	22	31	28	45	158

The concatenated version of the language was approximately two minutes long, varying slightly as a function of pause duration. After listening to the language for two minutes, participants were presented with a set of test trials. On each test trial, participants heard both a word (either diti or dapu) from the language, and a syllable combination formed across word boundaries (godi, or tibu), which we call a part-word. Test items were presented with no pauses between syllables.

#### Dimension-switching task

In this task, participants were presented with either a series of images, or a series of words. Each item in the series was surrounded by either a red or a blue border. The border indicated whether participants should rate the presented item as living/nonliving, or as large/small (the mapping between color and rating task was counterbalanced across participants).

Image stimuli were photographs with backgrounds removed adapted from prior published work (Moreno-Martínez and Montoro, 2012). Objects from this collection of images were balanced between categories by number of letters and syllables. The stimuli were presented within a bounding box of 720 pixels by 540 pixels. The average width of an image was 362 pixels (min: 35 px, max: 674 px, SD: 177 px) and a height of 334 pixels (min: 81 px, max: 684 px, SD: 148 px). Word stimuli labeled the same set of concepts as presented in the image stimuli, and were displayed with the first letter capitalized in black, Open Sans font, on a white background.

Table 3 lists the 12 images and words that were depicted. Red and blue borders were added to both image and word stimuli, creating a total of 24 stimuli (each of the 12 items with either a red or a blue border).

**Table 3:** List of 12 objects used in task-switching task, categorized into Living/Nonliving and Small/Large.

	Living	Nonliving
Small	Ant, Lemon, Rose	Dice, Fork, Pencil
Large	Tree, Dolphin, Cow	Chair, House, Bed

### Procedures

All participants completed the experiment remotely, through personally owned computers at times and places of their convenience. Access was restricted to computers (no phones or tablets were permitted), and the experiment was conducted via Gorilla, a web platform for experiments. All participants completed the experiment in English. Consent was collected and a sound check was conducted before any data were collected. Participants then completed the word segmentation task, followed by the Simon task, the audio and visual digit span tasks, and the N-back tasks in randomized order. Afterwards, participants completed questionnaires on language experience and demographics, as well as the dimension-switching task. All task instructions were presented visually on screen before the task began; participants were asked to press the spacebar to indicate that they understood the instructions and were ready to proceed.

#### Word segmentation task

Participants were randomly assigned to a cue version of the language (Convergent, Conflicting) and a pause duration (0 ms, 10 ms, 15 ms, 25 ms, 50 ms). In the Convergent Cue versions of the language, pauses occurred at statistically-defined word boundaries. In the Conflicting Cue versions of the language, pauses occurred between syllables within statistically defined-words (and thus, indicate different segmentation points than the statistical information). Note that at 0 ms, the Conflicting and Convergent Cue versions are identical, as the pauses do not occur (ie, they have a length of 0 ms).

After participants were randomly assigned to cue condition and pause duration, they listened to the appropriate version of the artificial language for approximately two minutes. After listening to this artificial language, participants were presented with eight test trials, in which they were asked to identify which item sounded more familiar to them. On each trial, participants heard two test items (one word, and one part-word), and were instructed to press the “1” key if they believed that the first item sounded more familiar to them, and the “2” key if they believed the second word was more familiar. Test item presentation order within and across trials was counterbalanced across participants.

#### Simon task

On each trial, the word “LEFT” or “RIGHT” was presented on either the left or right side of a fixation cross. Participants were instructed to click “Q” when the word “LEFT” appeared on the screen and “P” when the word “RIGHT” appeared, regardless of their location on the screen. The task consisted of 32 randomized trials with feedback. In each trial, the word “LEFT” or “RIGHT” was displayed for 900 ms, followed by a 500 ms pause before the next trial. Participants could respond at any time during the total 1,400 ms. Upon a correct response, the screen displayed “Correct,” covering the fixation cross. If the participant responded incorrectly or did not respond within the 1,400 ms, the screen displayed “Incorrect,” as well as a brief reiteration of the instructions. Feedback for both correct and incorrect trials were shown for 2,000 ms, either immediately after the response, or after the 1,400 ms maximum trial time, if the participant did not respond.

#### Audio and visual digit span tasks

A series of randomly generated numerical digits were presented one at a time. In the visual span, the digits were presented on the screen with a fixation cross in between each digit. In the audio digit span, the digits were read with a 1-s pause between each digit. Participants were instructed to focus on the fixation cross during the reading of the digits. The task was to memorize the digits in order and type them after they were presented. The trials began with a four-digit-trial (“1 2 3 4”), followed by 2 trials of each number of digits, from 4 to 9 digits, for a total of 13 trials.

#### N-back task (2-back)

A series of letters were displayed on the screen one at a time in a pseudo-randomized order. Participants were instructed to indicate whether the current letter was the same as the one that appeared two letters ago, by pressing either “F” or “J” on the keyboard. The key that indicated a match was counterbalanced between participants. The task was divided into three blocks of 10 trials each, with the instructions redisplayed on the screen between each block.

#### Language and demographics questionnaires

Participants completed the LEAP-Q (Marian et al., 2007) regarding their linguistic background and experience. Familiarity with a language was determined using 7 free-response questions regarding age and setting of acquisition. Proficiency and extent of use for each language with which the participant reported familiarity was assessed using three likert scales, and 13 true-false questions, for each language (up to 4). After completing questions about their linguistic background, participants were asked to provide general demographic information such as age and gender, as well as ethnicity and nationality.

#### Dimension-switching task

**Figure 1:**
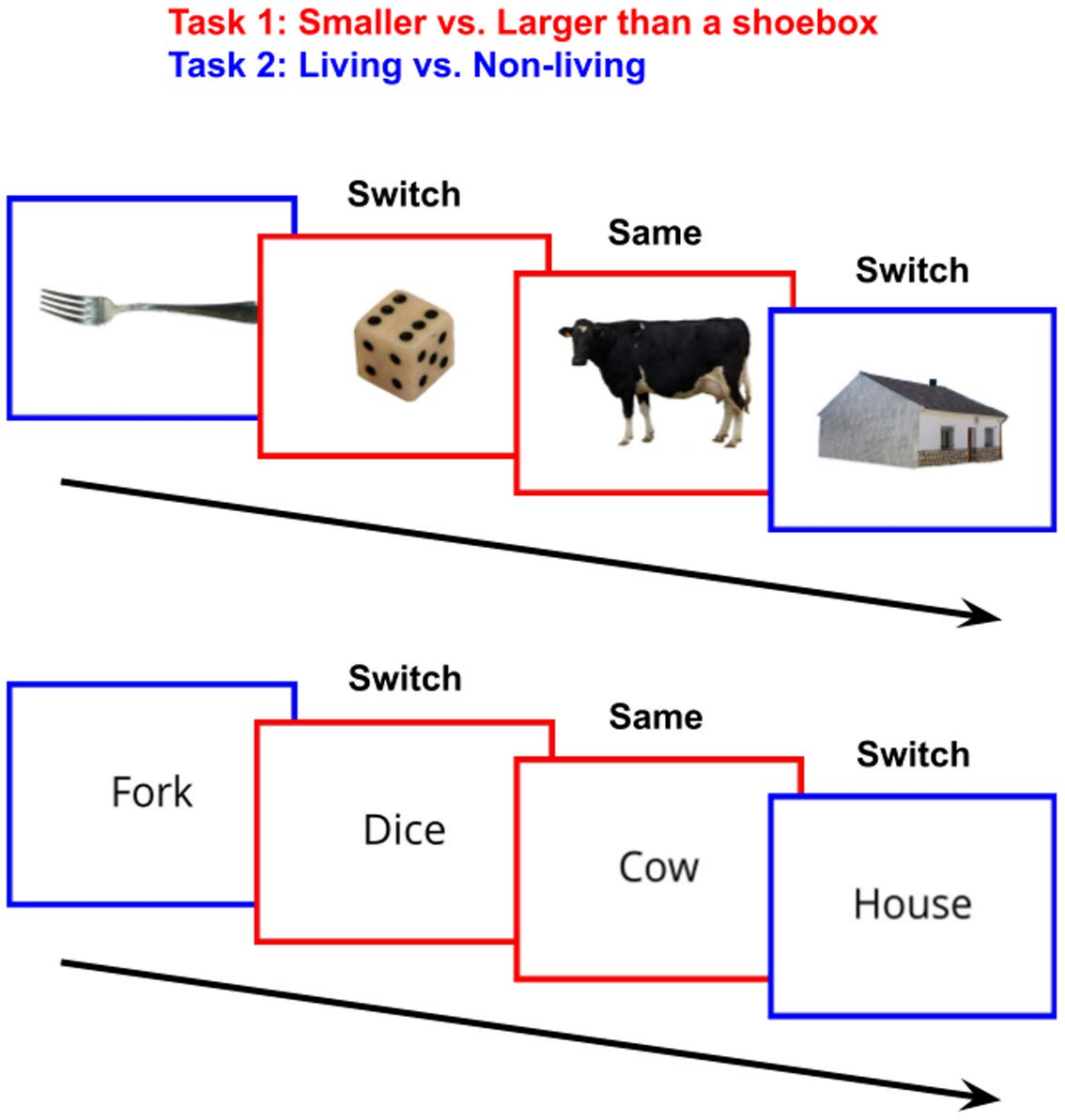
Task-switching procedure; In each trial, participants were shown an image or word stimulus surrounded by a blue or red border, which indicates the task to complete. When the task is the same as the task for the previous trial, the current trial is a “Same” trial. If it was the alternate task, the current trial is a “Switch” trial.

In the dimension-switching task, participants were presented with a stimulus (either an image or a word), surrounded by a solid colored border. The color of this border indicated the judgment the participants should make about the stimulus: whether it is living/nonliving, or larger/smaller than a shoebox (Figure 1). Each participant was randomly assigned to either the image condition (*N* = 102), or the lexical condition (*N* = 106).

The experiment consisted of 24 practice trials, in which each stimulus was shown once, followed by 120 test trials, in which each stimulus was shown a total of five times throughout the block, with no breaks. Each practice trial provided feedback (“Correct”/“Incorrect”), however participants were alerted after the practice block that trials would no longer provide feedback. Each stimulus was shown for 3,000 ms, during which the participant was able to respond, followed by 500 ms of feedback for the practice trials. Between each trial, a fixation cross was shown for 300 ms, with 100 ms before and after, totalling 500 ms between the end of a trial and the start of the next trial. All trials were randomized in order.

### Data analysis

#### Language proficiency score

**Figure 2:**
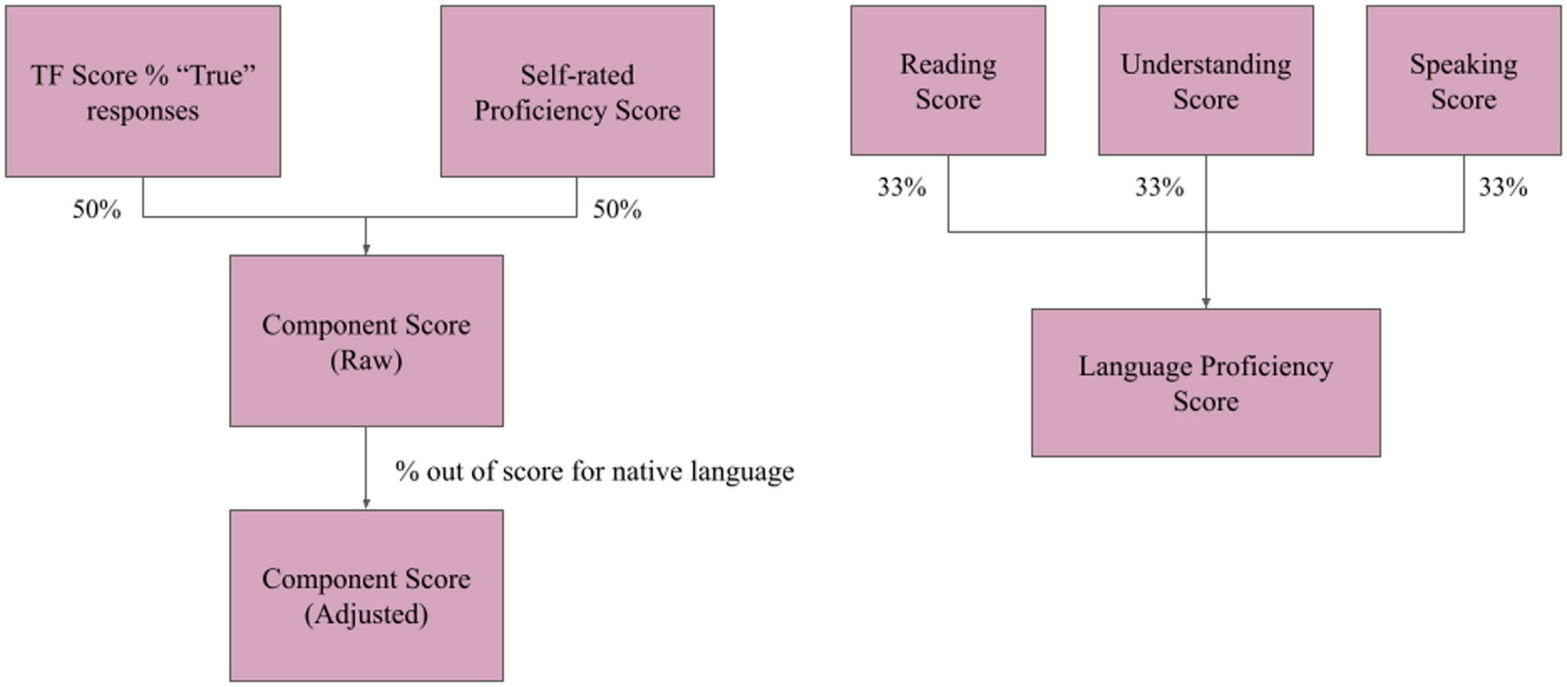
(Left) The component scores (reading, understanding, and speaking scores) were calculated by equal weighting of the True/False questionnaire responses and the self-rated proficiencies. These were normalized as a percentage out of the score for the participant’s native language. (Right) Language proficiency scores for each language a participant speaks were calculated by equal weighting of each of the component scores (reading, understanding, and speaking scores).

Each participant provided us with a self-report on the number (up to 4, in this sample) of languages with which they were familiar enough to use in social contexts. Additionally, to better reflect each participant’s overall language experience, we developed a continuous total language proficiency measure of multilingualism from the rest of the LEAP-Q responses. For each component (reading, understanding, or speaking) we calculated a component score based equally on the participants’ response to a series of true-false questions (the true-false score) and the self-rated proficiency score. For each of the languages spoken by the participant, a language proficiency score was calculated based on the component scores for that language, where each component was equally weighted (Figure 2). The total proficiency score is the sum of the language proficiency scores of all languages spoken by a participant. A total component proficiency score was also calculated, as the sum of the component proficiency scores of all the languages known by the participant.

While fluency in more languages provides a participant with the opportunity to generate a higher proficiency score (as reading/speaking/understanding questions are only asked about those languages that a participant initially reports familiarity with), it is not the case that number of languages perfectly predicts proficiency score, though these measures are highly correlated (*β* = 6.14, *R^2^* = 0.822, *p* < 0.001). While proficiency scores tend to increase as participants report familiarity with more languages, there are a number of exceptions to this general rule. For example, sometimes participants report being familiar with languages that they self-studied as a hobby, but acquired little real fluency with. Proficiency scores—which provide more detail about a participant’s daily use and experience with a language—help to differentiate those participants who are using languages regularly from those who have a more passing or superficial familiarity.

## Results

### Working memory

#### Audio digit span

For all participants, the average score on the digit span task was 6.90 items (SD = 1.29). For monolingual participants, this score was 7.00 (SD = 1.23). For multilingual participants, this score was 6.87 (SD *=* 1.31). This difference between monolingual and multilingual participants was not significant [*t*(206) = −0.62, *p* = 0.537].

A similar result held when we treated language proficiency as a continuous variable. Neither overall proficiency [*t*(206) = 0.23, *R^2^* = 0.000, *p =* 0.821], nor any of its components [reading (*p* = 0.889), speaking (*p* = 0.879), understanding (*p* = 0.716)], significantly predicted audio digit span.

#### Visual digit span

For all participants, the average score on the visual digit span task was 6.70 items (SD = 1.46). For monolingual participants, this score was 6.30 (SD = 1.20). For multilingual participants, this score was 6.82, (SD = 1.52). This difference between monolingual and multilingual participants was significant, (*t*(206) = 2.49, *p* = 0.013).

A similar result held when we treated language proficiency as a continuous variable. A regression analysis indicated that language proficiency significantly predicted scores in the visual digit span task (*t*(206) = 4.39, *R^2^* = 0.081, *p* < 0.001). All three components were also significant predictors (*p* < 0.001).

#### N-back task

For all participants, we calculated the proportion of correct trials. Across all participants, the proportion of correct trials was 74.9% (SD = 15.1). The proportion of correct trials was not significantly different between monolingual participants (*M* = 74.9%, SD = 14.5) and multilingual participants (*M* = 74.9%, SD = 15.3; *t*(206) = 0.02, *p* = 0.984). When we treated language proficiency as a continuous variable, we found no evidence of a relationship between proficiency and the proportion of correct trials (*t*(206) = 0.64, *R^2^* = 0.002, *p* = 0.526), or between any of its components and the proportion of correct trials (all *p*-values are greater than 0.250).

An analysis of reaction time found no evidence of differences in performance between monolingual and multilingual participants (*t*(206) = −0.34, *p* = 0.731), or evidence of a relationship between proficiency score and reaction time (*t*(206) = −0.19, *R^2^* = 0.000, *p* = 0.851).

#### Summary

Consistent with prior results, our sample indicates that working memory performance is similar between monolingual and multilingual participants. The exception to this is in the visual digit span task, where participants who reported familiarity with multiple languages showed better performance than those who knew only English. It is somewhat counterintuitive that language background would have greater influence on a visual memory as opposed to auditory memory, given the auditory nature of the languages with which participants reported familiarity. As such, this result may be anomalous or spurious. It is at least inconsistent with meta-analytic work indicating that any difference in working memory tasks is most likely to be found in verbal working memory tasks (Monnier et al., 2022).

More generally, these results suggest that our monolingual sample is comparable to our multilingual sample. Even for the visual working memory task where there is a significant difference between groups, the effect size (Cohen’s *d* = 0.37) is small. There is little reason to believe that these groups should show large differences in their performance on cognitive tasks. Instead, they appear to be relatively comparable, as would be expected of a group of participants who have all been selected for admission into a prestigious private university.

### Word segmentation and the Simon task

#### Word segmentation

Across all cue conditions and pause durations, the average number of correct trials out of eight total trials in the word segmentation task was 4.18 (SD = 2.75). To assess the effect of cue conflict in word segmentation, we performed a regression analysis with cue version (Convergent or Conflicting) and pause length (0, 10, 15, 25, or 50 ms) as predictors. There was a significant main effect of cue condition [*t*(198) = −6.63, *p* < 0.001]. On average, participants’ ability to identify statistically defined words (i.e., the number of “correct responses”) was much higher in the Convergent condition (*M =* 6.39, SD *=* 1.66*)* than in the Conflicting condition (*M* = 2.15, SD = 1.82). This suggests that our manipulation of cue conflict was at least somewhat effective, as participants identified (statistically defined) words more easily in situations where pause cues aligned with those words than in situations where pause cues occurred in the middle of statistically-defined words in the input stream.

There was no significant main effect of pause length, nor were any pairwise comparisons between pause duration significant (all *p*-values greater than 0.250). For example, the participants’ average performance at 0 ms (*M* = 4.26, SD = 2.51) was quite similar to their average performance at the 50 ms pause duration (*M* = 5.10, SD = 2.63). While it is difficult to interpret null effects, the lack of performance difference across pause durations is consistent with the possibility that pause cues have relatively symmetric effect. That is, they improve performance in the convergent cue languages as much as they impair performance in the conflicting cue languages, such that there is no overall effect of pause duration on performance.

As we predicted, there was an interaction between cue condition and pause length such that participants’ performance was worse in the Conflicting condition (*M* = 1.79, SD = 2.04) than in the Convergent condition (*M* = 6.72, SD = 1.56) at the 50 ms pause length [*t*(198) = −1.81, *p* = 0.072], but not at any other pause lengths. This result suggests that at pause lengths of less than 50 ms, participants do not weigh the pause cue as heavily, consistent with prior results for English speakers (Weiss et al., 2010).

Language background had no apparent effect on performance in the word segmentation task, regardless of whether we considered language as a categorical factor, or treated it as a continuous variable. Across all levels of language familiarity and proficiency, there were no significant main effects of language background, nor any interactions with cue condition or pause duration.

Nevertheless, these results indicate that there is significant cue competition between statistical information and pauses at 50 ms (but not 0 ms). Therefore, we should expect that if performance in the word segmentation task is tapping into the same kind of conflict resolution processes implicated in executive function, then performance in the Simon task might be correlated with performance in the word segmentation task at 50 ms pause duration. We should not expect any correlation with performance in the 0 ms version of the word segmentation task, as there is no conflict to resolve—no pauses—in this version of the language. At pause durations between 0 and 50 ms, we might expect somewhat intermediate values of conflict resolution, but for the sake of expositional clarity, we will focus on the 0 ms condition (minimal competition to resolve) and the 50 ms condition (maximal competition to resolve) to investigate as predictors of performance in the Simon task.

#### Simon task

In the Simon task, participants are presented with Congruent trials, in which the presented stimulus (the word “LEFT” or the word “RIGHT”) and the appropriate response are on the same side of the screen, and Incongruent trials, in which the stimulus is on the opposite side of the screen from the appropriate response. The Simon Effect is derived by comparing the average reaction time of Congruent trials to the average reaction time of Incongruent trials (which will almost always be longer, on average). A small Simon Effect means that reaction time to Incongruent trials is almost as fast as reaction time to Congruent trials, and is a sign of effective conflict resolution. A large Simon Effect means that reaction time to Incongruent trials is much slower than reaction time to Congruent trials, and is a sign of difficulty with conflict resolution.

To calculate a Simon Effect for each participant, we first removed incorrect trials from the dataset (total accuracy was as 93.90%, SD = 6.66, such that errors represented less than 7% of total trials). Of the remaining correct trials, as expected, participants were slower to respond to Incongruent trials; the average reaction time for Congruent trials was 567.70 ms (SD = 77.28), while the average reaction time for Incongruent trials was 590.72 (SD = 76.21). For each participant, we calculated a Simon Effect by subtracting their average reaction time on Congruent trials from their average reaction time on Incongruent trials.

Over all participants, the average magnitude of the Simon Effect was 23.03 ms (SD = 50.77). The magnitude of the Simon Effect was not different between monolingual and multilingual participants [*t*(206) = 0.69, *p* = 0.489], nor was it in the direction predicted by a bilingual cognitive advantage, as monolinguals had a numerically (though not significantly) smaller Simon Effect (*M* = 22.7, SD = 54.3) than their multilingual peers (*M* = 23.5, SD = 45.9). The continuous language proficiency score, on the other hand, was a marginally significant predictor of the magnitude of the Simon Effect [*t*(206) = 1.68, *R^2^* = 0.013, *p* = 0.094]. Our regression analysis indicated that higher language proficiency scores correspond to a numerically larger Simon Effect (*β* = 1.01). When breaking the proficiency score into components, reading proficiency [*t*(206) = 1.83, *R*^2^ = 0.016, *p* = 0.069] and understanding proficiency [*t*(206) = 1.68, *R*^2^ = 0.013, *p* = 0.087] were marginally significant predictors of Simon Effect magnitude, while speaking proficiency was not [*t*(206) = 1.72, *R^2^* = 0.014, *p* = 0.202].

In addition to the magnitude of the Simon Effect, we also investigated, as suggested by a thoughtful reviewer, the overall reaction time (RT) in congruent trials, in incongruent trials, and averaged over all trials. In congruent trials, the average accuracy among all participants was 96.06% (SD = 6.09) and average RT was 567.70 ms (SD = 77.28). There was no significant difference in congruent RT between monolinguals (*M* = 557.63, SD = 83.60) and multilinguals (*M* = 570.88, SD = 75.16; *t*(206) = 0.99, *p* = 0.322). A similar (lack of) relationship held when using the continuous language proficiency score to predict reaction times on congruent trials [*t*(206) = 1.61, *R*^2^ = 0.014, *p* = 0.108]. Additionally, none of the components of the proficiency score significantly predicted congruent RT.

For incongruent trials, the average accuracy was 91.74% (SD = 9.59) and the average RT was 590.72 (SD = 76.21). When treating language experience as a categorical variable, there was no significant difference in incongruent RT between monolinguals (*M* = 575.97, SD = 79.79) and multilinguals [*M* = 595.39, SD = 74.69; *t*(206) = 1.51, *p* = 0.132]. However, the continuous proficiency measure was a significant predictor of incongruent RT such that greater proficiency corresponded with longer RT [*t*(206) = 2.67, *R*^2^ = 0.038, *p* = 0.008]. All three components of proficiency also significantly predicted incongruent RT (*p* = 0.012 for reading, *p* = 0.013 for speaking, and *p* = 0.009 for understanding). While this is not an effect we predicted, we do note that it is at least consistent with the claim that a continuous measure of linguistic experience may be a more sensitive measure than a categorical sorting.

Across all trials, the average RT was 578.87 (SD = 72.69). The relationship with language background showed a similar pattern as incongruent RT. The overall RT was not significantly different between monolinguals (*M* = 566.59, SD = 76.88) and multilinguals [*M* = 582.76, SD = 71.12; *t*(206) = 1.31, *p* = 0.192]. At the same time, greater proficiency significantly predicted longer overall RT [*t*(206) = 2.22, *R*^2^ = 0.027, *p* = 0.028], and all three of its components were also significant predictors (*p* = 0.034 for reading, *p* = 0.041 for speaking, and *p* = 0.030 for understanding). The significant effect seen while aggregating across all trials was largely driven by the difference in RT in Incongruent trials, though the (non-significant) trend in the Congruent trials appears to be in the same direction.

These results are not consistent with a multilingual advantage in conflict resolution. While categorical groupings of our participants indicated no difference in performance between monolingual and multilingual participants, our continuous measure of language background indicated that increasing language proficiency predicted a larger Simon Effect. That is, the more behavioral evidence a participant reported of using multiple languages, the slower they were to resolve conflict and produce a response in incongruent trials.

#### Relations between word segmentation and the Simon effect

To the extent that conflict resolution via attentional control is a domain general or task general ability, we should expect to see that participants who are good at conflict resolution in one task should also be successful in other tasks that measure conflict resolution. In particular, prior research has suggested that the Simon Effect might be related to conflict resolution in word segmentation, in both monolingual (Weiss et al., 2010) and bilingual (Bartolotti et al., 2011) populations. To determine whether we replicated this claim, we used a regression analysis to see if the Simon Effect predicted word segmentation accuracy, as a function of cue conflict and pause duration.

**Figure 3:**
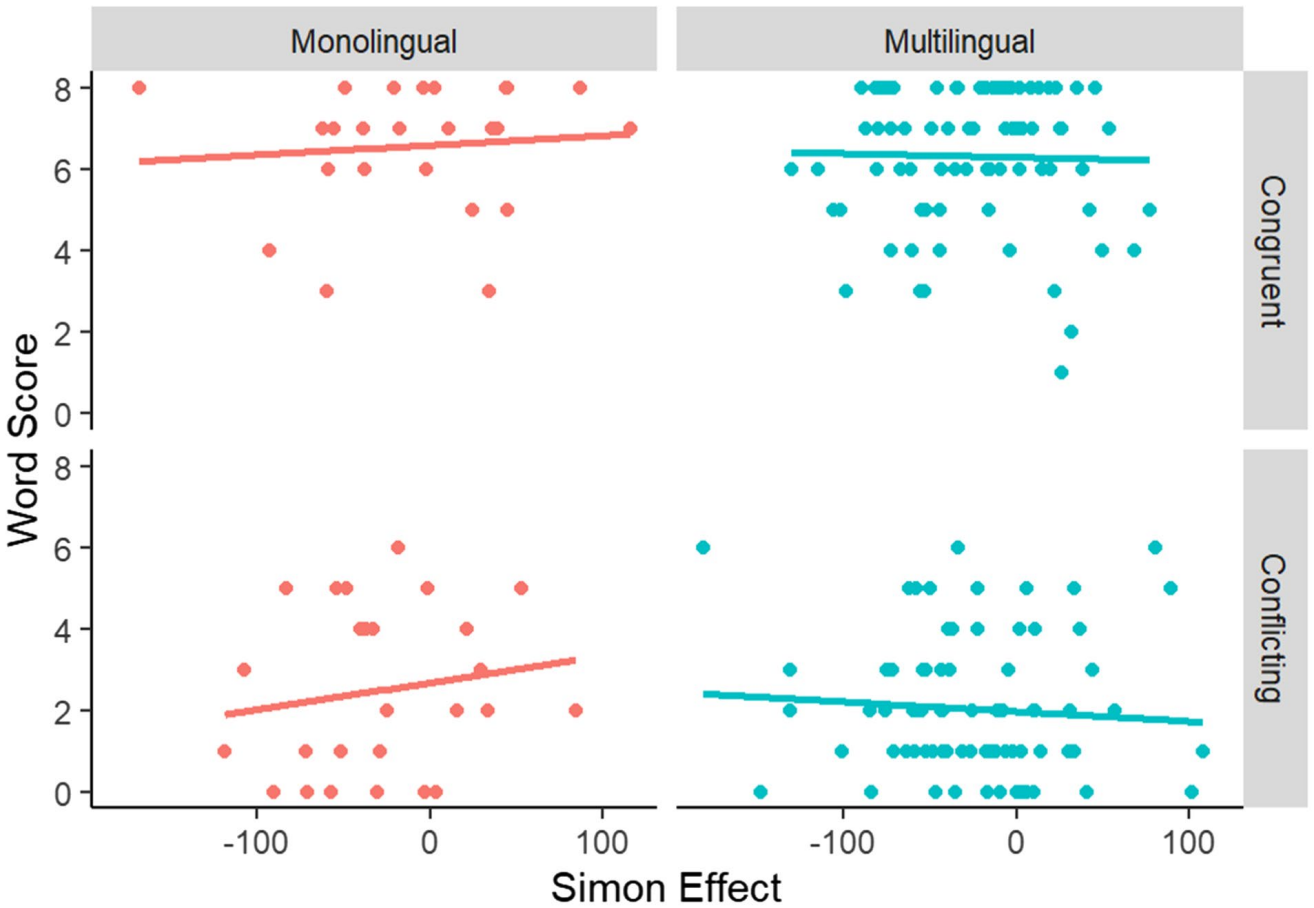
Relationship between the Simon Effect (ms) and performance on the Word Segmentation task (out of 8 questions), by multilingualism and word segmentation cue version.

Our analysis indicated that the magnitude of the Simon Effect was not predictive of performance on the segmentation task. This was true when considering all participants contributing data [*t*(206) = −0.54, *R*^2^ = 0.001, *p* = 0.590], the monolingual subgroup only [*t*(206) = −0.73, *R*^2^ = 0.005, *p* = 0.467], or the multilingual subgroup only [*t*(206) = 0.09, *R*^2^ = 0.000, *p* = 0.932; see Figure 3]. Similarly, regression analysis indicated that continuous language proficiency did not moderate the relation between Simon Effect and word segmentation performance.

**Figure 4:**
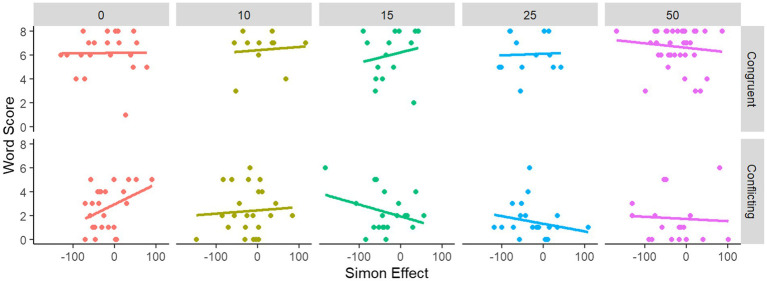
Relationship between the Simon Effect (ms) and performance on the Word Segmentation task (out of 8 questions), by pause length and word segmentation cue version.

As discussed above (see “Word Segmentation”), pauses become a more powerful cue as their duration increases, and at 50 ms there appears to be maximum conflict between pause cues and statistical cues. By contrast, at 0 ms, there is minimal conflict, because pauses (definitionally) do not occur. Therefore, we assessed whether pause duration moderated the relationship between Simon score and word segmentation task. Our analyses indicated that there was no systematic relationship between these variables (see Figure 4). Indeed, the relationship between Simon task and word segmentation was stronger at 0 ms (where there is no possible pause-related conflict resolution in the word segmentation task) than at 50 ms.

#### Summary

These results provide little evidence to support claims for a multilingual advantage. Our primary hypothesis, that conflict resolution as measured in the Simon task would be predictive of conflict resolution in a linguistic task—word segmentation in the face of conflicting cues—was not supported. This fails to replicate prior work observing such a relationship in both monolingual (Weiss et al., 2010) and multilingual (Bartolotti et al., 2011) populations, so our failure to find evidence of the relation in either group is noteworthy.

One possibility that may explain our failure is that while our manipulation of pause duration (50 ms) was identical to that used in past studies, this pause duration may have been more (or less) salient to our participants than has been the case in prior studies. In particular, prior studies assessing the relation between segmentation performance and Simon Effect have used segmentation languages that are more complex than ours in a variety of ways, such as having more words, longer words, or more challenging perceptual features than the relatively simple 4 bisyllabic word language used in this study.

At the same time as we failed to find support for our primary hypothesis, we also found evidence that was directly contradictory to claims of a multilingual advantage: multilingual participants appeared to perform worse in the Simon task. This is inconsistent with several prior published results suggesting a bilingual or multilingual advantage for conflict resolution (e.g., Bialystok et al., 2004). These results are not totally unprecedented, as not all investigators have found a multilingual advantage in conflict resolution (e.g., Morton and Harper, 2007). Moreover, it is worth noting that our results indicating a “monolingual advantage” were only significant when we treated language background as a continuous measure.

### Dimension switching

In the Dimension-Switching task, participants are presented with a stimulus (either a word, or an image), and instructed by a colored border around the stimulus to make a particular judgment (living/non-living or large/small) about the stimulus. On trials where participants are making a different judgment than the one they made on the prior trial, their reaction should be somewhat slower than if they are repeating the same judgment from the previous trial. This “switch cost” is the primary dependent variable of interest in the task. Prior work suggests that multilingual speakers should have a smaller switch cost than monolingual speakers (e.g., Prior and MacWhinney, 2010).

**Figure 5:**
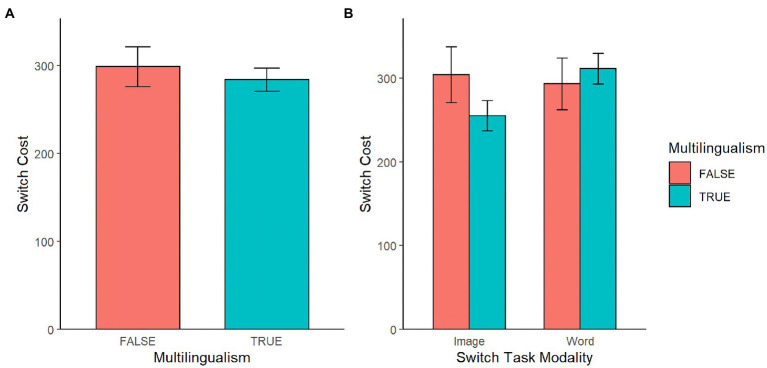
**(A)** Switch task performance by multilingualism; and **(B)** switch task performance by multilingualism and switch task modality.

**Figure 6:**
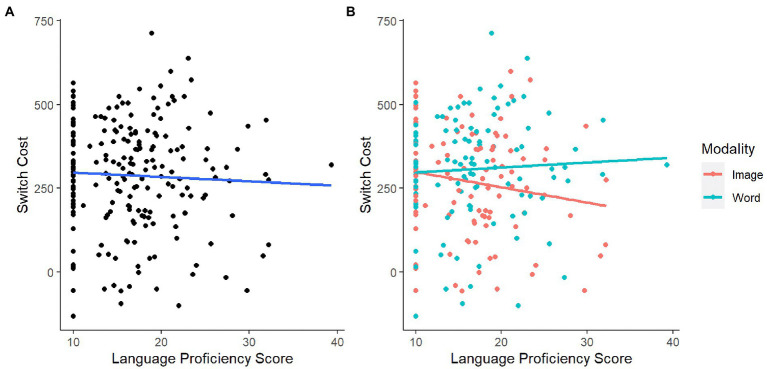
**(A)** Switch task performance by language proficiency; **(B)** switch task performance by language proficiency and switch task modality.

Performance was similar across different language backgrounds. Multilingual participants had numerically smaller switch costs (*M* = 284.33, SD = 163.91) compared to monolingual participants (*M* = 299.06, SD = 160.00; see panel A in Figure 5). Consistent with this observation, regression analysis indicated that participants with higher language proficiency score had lower switch cost (*β* = −1.31; see panel A in Figure 6). However, neither the monolingual-multilingual dichotomy [*t*(204) = −1.27 *p* = 0.205] nor the continuous language proficiency [*t*(204) = −1.52, *p* = 0.131] significantly predicted switch cost.

#### Dimension-switching and linguistic stimuli

**Figure 7:**
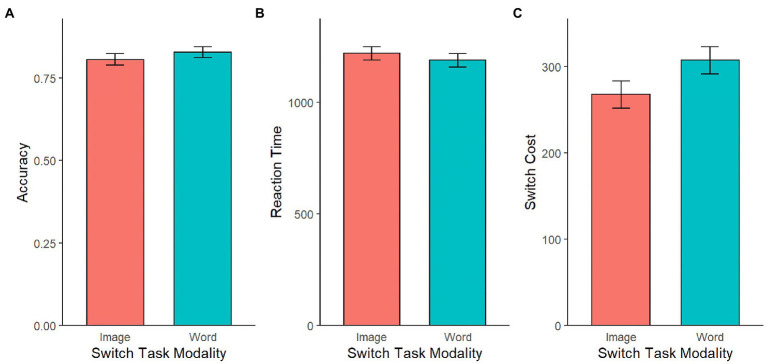
Effect of switch task modality on accuracy **(A)**, reaction time **(B)**, and switch cost **(C)**. There were no significant differences in accuracy, reaction time, or switch cost across the two task versions.

Of interest to us is whether switch cost is more closely related to language background when the dimension-switching task uses explicitly linguistic stimuli (orthographic words) or when it uses stimuli that are less directly tied to linguistic representations (photorealistic visual images). We instantiated this modality difference as a between-subjects variable. Overall performance was very similar between the orthographic word and photographic images versions of the task (see Figure 7). Across the two versions of the task, there is no overall difference in accuracy [*t*(206) = 0.94, *p* = 0.350] or reaction time [*t*(206) = −0.73, *p* = 0.464]. Note, however, that there is some hint of a difference in switch costs across modality, as switch costs were somewhat greater with lexical stimuli, a difference that was marginally significant [*t*(206) = 1.76, *p* = 0.079].

To assess whether language background was differentially related to switch cost as a function of stimulus modality, we used linear regression with three predictors: language background, stimulus modality, and the interaction term. We fit two models, one treating language background as a binary classification (monolingual, multilingual) and one using the continuous proficiency score. Stimulus modality did not emerge as a significant predictor, regardless of whether language background is included as a categorical variable [*t*(204) = −0.23, *p* = 0.816] or a continuous variable [*t*(204) = −0.90, *p* = 0.367]. This null effect is consistent with the similar performance across the pictographic and orthographic versions of the task described above.

The same regression analyses indicated no significant interaction between stimulus modality and language background. In the image version of the task, the switch cost was numerically smaller for multilingual participants (*M* = 255.06, SD = 156.73) than for monolingual participants (*M* = 304.24, SD = 170.73); in the word version, the switch cost was higher for multilingual participants (*M* = 311.46, SD = 166.68) than for monolingual participants (*M* = 293.44, SD = 166.68; see panel B in Figure 5). Consistent with these descriptions of the data, our regression analysis showed that language background predicted lower switch cost in the image version (*β* = −4.56), and predicted higher switch cost in the word version (*β* = 1.47; see panel B in Figure 6). However, these interactions between stimulus modality and language background were not found to be significant in either model [*t*(204) = 1.26, *p* = 0.208 with categorical, *t*(204) = 1.57, *p* = 0.119 with continuous].

#### Dimension-switching and word segmentation

As we noted in the introduction, transfer across tasks is more likely when those tasks are similar to some extent. Because the dimension-switching task relied upon the linguistic nature of the stimuli, we were prompted by a helpful comment from reviewers to investigate the relation between the dimension-switching task and word segmentation task, which is also putatively linguistic in nature. Switch cost was not a significant predictor of performance on the word segmentation task [*t*(206) = 0.93, *R*^2^ = 0.004, *p* = 0.355]. When including language experience in the model, none of the main effects or interactions were significant (all *p*-values greater than 0.200), regardless of whether the binary classification of monolinguals and multilinguals or the continuous language proficiency score (composite or any of the components) was used.

#### Summary

As with the relationship between Simon Effect and word segmentation, our investigation of the relationship between dimension switching and stimulus modality did not support our hypotheses. We did not find that language background more strongly predicted performance with linguistic stimuli than performance with pictorial stimuli. Indeed, while none of our effects were significant, the relationship that seems most robust is between linguistic background and performance in a pictorial version of the task, where multilingual speakers show most robust evidence of an advantage with pictorial stimuli, while monolingual participants appear to have an advantage for orthographic stimuli. These results, while potentially suggestive of patterns that might be revealed by a larger sample or more sensitive design, are the opposite of what we predicted. Similarly, we failed to replicate prior results that multilingual participants have an advantage in dimension switching (e.g., Prior and MacWhinney, 2010; Wiseheart et al., 2016).

### Language dominance

The primary novel hypothesis that motivated this research (in addition to the desire to attempt a conceptual replication of Weiss et al., 2010) was that the “distance of transfer” would predict the degree to which multilingual participants show a difference in executive function skills from monolingual participants. That is, we predicted that monolingual and multilingual participants would perform in a relatively similar fashion for non-linguistic tasks, and less similarly in linguistic tasks, a prediction we attempted to directly assess with our manipulation of cue presentation in the task switching task.

However, our design may work against our ability to detect such differences. Specifically, we conflated the “linguistic” nature of our stimuli with the English language. It might be the case that multilingual speakers are indeed more successful in executive function tasks that involve linguistic stimuli or processes. But because all of our linguistic stimuli are instantiated in English, our multilingual participants’ advantage may be masked by a general advantage for monolingual speakers when tested in their native language.

As suggested during the review process, we sought to assess the plausibility of this alternative explanation by more thoroughly investigating how participants’ self-reported language dominance (that is, the language with which they feel most comfortable, fluent, and practiced) was related to their task performance. To the extent that a multilingual advantage is masked by a general multilingual disadvantage (compared to monolingual speakers) with English stimuli, we might expect that this disadvantage would be less severe for those multilingual participants who report that English is their dominant language. Conversely, if multilingual participants show relatively similar performance, regardless of their language dominance, this would suggest that an English-mask of the multilingual advantage is less plausible (though certainly not impossible). Additionally, a more extensive investigation of language background and language dominance may provide us with some insight about the generalizability of our results.

As such, the analyses below investigate performance in our 158 multilingual participants, sorted by whether they report English as their dominant language (120 participants), or report that some other language (38 participants) is dominant in their daily use. We explored whether this sorting predicts differences in the Simon task, which is the task for which we have the most data for each participant, and thus presumably the task most sensitive to individual differences. Additionally, we analyzed data from the Dimension Switching task, which we explicitly designed to test the effect of linguistic stimuli.

#### Simon task

In terms of accuracy, the 120 English-dominant participants (*M* = 0.937, SD = 0.072) and the 38 Other-dominant participants (*M* = 0.938, SD = 0.067) showed similar performances (*p* = 0.984). This was also true when analyzing only congruent (*p* = 0.867) or only incongruent trials (*p* = 0.888).

Overall reaction times (RT) of English-dominant participants (*M* = 578.18, SD = 72.07) were not significantly different (*p* = 0.14) than the reaction times of Other-dominant participants (*M* = 597.22, SD = 66.88). A linear regression with multilingualism as a continuous variable also showed that differences between the English-dominant and Other-dominant participants were not significant (*p* = 0.388). These results held for analyses within congruent and incongruent trials, such that both t-tests and linear regressions incorporating the multilingualism continuous variable resulted in non-significant effects of language-dominance on RT (all *p* > 0.10). The only marginally significant relation found was between multilingualism as a continuous score and RT specifically in incongruent trials [*t*(154) = 1.82, *p* = 0.070]. This relationship was not significant in congruent trials and over all trials averaged. Note that these exploratory analyses are not corrected for multiple comparisons (to allow for greatest sensitivity to possible patterns of interest), so marginal significance and even significant effects should be interpreted cautiously.

English-dominant participants showed a numerically smaller Simon Effect (*M* = 24.28, SD = 48.59) than Other-dominant participants (*M* = 25.24, SD = 51.29). However, this difference was not significant (*p* = 0.920). A linear regression with multilingualism as a continuous variable also indicated non-significant effects (*p* = 0.409).

#### Dimension switching

English-dominant participants had a mean accuracy of 0.799 (SD = 0.181), and Other-dominant participants had a mean accuracy of 0.849 (SD = 0.158). This difference was not significant (*p* = 0.110). Analysis of accuracy in the image version of the task between English-dominant (*N* = 59) participants (*M* = 0.798, SD = 0.172) and Other-dominant (*N* = 17) participants (*M* = 0.796, SD = 0.191) also showed no significant differences (*p* = 0.966).

However, in the word version of the task, Other-dominant participants (*N* = 21) showed higher accuracy (*M* = 0.892, SD = 0.112) than English-dominant (*N* = 61) participants (*M* = 0.800, SD = 0.192). This effect is significant [*t*(80) = 2.59, *p* = 0.011], but counterintuitive, as we would not expect English-dominance to be associated with lower accuracy on a task presented in English. The effect is also potentially consistent with a speed-accuracy tradeoff, meaning that this result cannot be interpreted in isolation from data about reaction time.

Overall, reaction times (RT) of English-dominant participants (*M* = 1187.50, SD = 323.15, *N* = 120) and those of Other-dominant participants (*M* = 1233.20, SD = 305.44, *N* = 38) did not significantly differ (*p* = 0.434). Upon analyzing differences between the English-dominant and non-English dominant groups within the image and word task modalities, we found that there were no significant differences in either modality (*p* = 0.979 for image version, *p* = 0.287 for word version). The lack of differences in reaction time is not consistent with the possibility that a speed-accuracy tradeoff explains the Other-dominant participants’ surprising advantage in accuracy on the lexical version of the task (though the lack of a significant effect certainly does not *rule out* the possibility of a speed-accuracy tradeoff).

Lastly, we analyzed differences in switch cost across participants. Recall that the switch cost is the primary dependent variable of interest in the Dimension-Switching paradigm; a low switch cost indicates stronger executive function, while a high switch cost indicates difficulty with the demands of the task. Overall, there was no significant difference (*p* = 0.145) in the switch cost between English-dominant participants (*M* = 273.19, SD = 161.07, *N* = 120) and Other-dominant participants (*M* = 319.52, SD = 169.96, *N* = 38). Similarly, the switch costs in the image version of the task showed no significant difference (*p =* 0.715) between the English-dominant participants (*M* = 250.84, SD = 146.97, *N* = 59) and the Other-dominant participants (*M* = 269.71, SD = 191.20, *N* = 17).

However, there was a marginally significant difference found in the lexical version of the task as a function of language dominance [*t*(80) = 1.67, *p* = 0.098]. Here, English-dominant participants (*M* = 294.81, SD = 172.08, *N* = 61) showed lower switch costs than Other-dominant participants (*M* = 359.84, SD = 142.74, *N* = 21). One could interpret this result as partially consistent with the hypothesis that (a subset of) multilingual speakers show a selective advantage on lexically instantiated executive function tasks. But even here, it should be noted that our English-dominant multilinguals’ switch cost on the task is virtually identical to that of monolingual participants (*M* = 293.44, SD = 166.68) in the lexical version of the dimension-switching task. Therefore, these results may indicate a selective disadvantage for Other-dominant multilingual participants in the lexical version of the task switching task. This result could suggest that engaging executive function is more challenging in non-dominant language contexts.

#### Summary

In the Simon task, our results indicate that participants’ language dominance had little effect on performance. This is consistent with the more general lack of evidence across our tasks, with the hypothesis that language background is related to performance in our executive function tasks. Interestingly, in the Dimension-Switching task, language dominance was related to both accuracy and the magnitude of switch cost. Due to the exploratory nature of these analyses, these results should be interpreted cautiously. But they are consistent with the claim that some degree of executive function performance may be masked or inhibited by the (English) linguistic nature of some of our stimuli.

## Discussion

At some level, it is inarguable that experience with multiple languages shapes the cognitive system of the people who speak them. An English speaker automatically connects the word “cat” to their representation of a whiskered mammal that purrs. An English-Spanish bilingual also does so for the word “gato,” in a way that the monolingual English speaker does not. The learning challenges and processes in a multilingual environment are different than those in a monolingual environment (e.g., Byers-Heinlein and Fennell, 2014; Singh, 2021). As such, our question is not “does experience with multiple languages change cognition,” but rather, what is the extent of those changes? The minimalist stance, consistent with a modular view of cognition (Fodor, 1983), is that experience with language only influences linguistic processing (e.g., Goldsmith and Morton, 2018; Dick et al., 2019). The maximalist view is that lifelong experience with multilingualism provides a domain-general strengthening of attentional processes associated with executive function, especially selection of relevant information, suppression of irrelevant information, and conflict resolution (e.g., Craik et al., 2010; Prior and MacWhinney, 2010; Bialystok, 2017). Or, to put it in terms borrowed from cognitive science, the central question is whether language experience “transfers” to only relatively close tasks, as is often seen with laboratory investigations of learning (e.g., Sala and Gobet, 2017). The alternative possibility is that language—perhaps due to our extensive experience with it, or its centrality to cognition—serves as a basis for far transfer, and has an influence that can be felt in a wide variety of different tasks, even those that are only minimally related to language (except by virtue of sharing some common underlying process).

Recent surveys of the field and meta-analyses (e.g., Donnelly et al., 2019; Vīnerte and Sabourin, 2019; Gunnerud et al., 2020; Paap et al., 2020; de Bruin et al., 2021) make a compelling case that both the maximalist position—of essentially unlimited far transfer to a variety of executive function tasks—and the minimalist position—expecting no transfer outside of linguistic tasks—are all but unsustainable in their purest forms. Rather than a consistent pattern of success (or failure) in transfer to cognitive tasks, the literature presents us a “mixed bag” of findings, with some converging replications of a multilingual advantage (e.g., Anderson et al., 2020a; Brini et al., 2020), several inconsistent or small effects (e.g., Lukasik et al., 2018; Paap et al., 2018), and even contradictory evidence of monolingual advantages in cognitive performance (e.g., Paap and Greenberg, 2013; Nichols et al., 2020). This presents us with the challenge of determining, not whether or not a multilingual advantage exists, but what are the modulating factors or constraints that determine when differences will exist across populations (van den Noort et al., 2019). This determination, however, is challenging given that the heterogeneity in multilingual participants and cognitive assessments provides a multitude of degrees of freedom that make falsification challenging (for discussion, see Struys et al., 2018). In such a situation, the value of theoretical frameworks as sources of falsifiable predictions is especially high (for discussion, see de Bruin et al., 2021).

One such theoretical perspective that has been advanced is a neuroscientific one (e.g., Vīnerte and Sabourin, 2019). This should be distinguished from the use of brain-based dependent variables, such as ERP or FMRI. Perhaps unsurprisingly, research efforts with these neural measures have produced the same type of mixed results as research efforts with more traditional behavioral measures (e.g., Leivada et al., 2021). Rather, we use the term neuroscientific theoretical perspective to mean research endeavors whose hypotheses are informed by an understanding of the structural, functional, and network characteristics of the brain (e.g., Hernandez et al., 2019; Del Maschio et al., 2020). For example, the Adaptive Control Hypothesis (Green and Abutalebi, 2013; Abutalebi and Green, 2016) generates predictions about the brain regions and cognitive processes that should be impacted by multilingual experience as a function of the dimensions on which those languages overlap, complement, and interfere with each other, and how those dimensions relate to known neural networks. While the Adaptive Control Hypothesis and other neurally inspired frameworks have inspired much productive research, a preliminary conclusion is that these theories are also incomplete, or fail to explain aspects of the data (for discussion, see Kałamała et al., 2020; Paap et al., 2021).

We propose that an embodiment perspective may enrich or complement the neuroscientific perspective, and other perspectives, and generate explanations and predictions about the relation between linguistic experience and cognitive processes. In particular, we hypothesized that multilingual participants would be more likely to show transfer from linguistic experience to executive control tasks (and thus, an advantage over monolingual participants) when the tasks prompted participants to engage in the same kinds of representations or processes that they engage in naturalistic language use. From this perspective, one reason why the prior literature on multilingual differences in executive control is mixed is that some tasks are much more likely to incorporate or evoke linguistic stimuli, which should make them more likely to detect differences between multilingual and monolingual participants.

Admittedly, the data that we generated to investigate predictions arising from an embodiment perspective are not tremendously consistent with our hypotheses. As noted in the results section, we repeatedly failed to support the hypothesis of a multilingual advantage in executive function—and indeed, even found evidence of a monolingual advantage in the Simon task—nor did we find evidence consistent with the claim that the linguistic nature of a task predicts the degree of multilingual advantage. However, we would like to suggest that these results should not be read as an indictment of the embodiment perspective. Instead, we believe that on further reflection, these results reflect essential limitations of our methodology, limitations that can be ameliorated by a more thorough integration of the embodiment perspective into this work.

First, some of the tasks that we chose to use may not have been sensitive to the individual differences between participants that we hoped to detect. In particular, the word segmentation task is likely to be a poor source of information about individual differences (e.g., Erickson et al., 2016; Siegelman et al., 2017a). This is not to say that the word segmentation task is uninformative. However, it is plausibly the case that the word segmentation task is informative about the differences between groups (such as monolingual and multilingual speakers) but not sensitive to individual differences among participants (for discussion, see Siegelman et al., 2017b). Because our design was aimed at assessing the relation between an individual’s Simon Effect and that same individual’s performance in the word segmentation task, we are focused on a level of analysis where the word segmentation task may be minimally sensitive.

A second limitation of our methodology is that we recruited a set of participants who are fairly heterogeneous in their language background and use, and at the same time—by virtue of their selection into an academically rigorous private university—not representative of the range of variation in cognitive performance and educational background seen in the population at large. In particular, while we found evidence that treating language background as a continuous variable can be informative, it is likely the case that our analyses overlook some of the continuous dimensions that are likely to shape the cognitive impact of language use, in particular age of acquisition and the degree of overlap or similarity between the languages. From an embodiment perspective, these questions are crucial, as they shape the way participants approach the task of language acquisition and use, which—in turn—should shape the kinds of tasks to which they transfer that experience. Indeed, the very heterogeneity of language backgrounds, and potential differences in participant populations, has led some to contemplate the possibility that the research question may not be tenable (e.g., Paap et al., 2014; Hartsuiker, 2015, though see Leivada et al., 2021). At the very least, it seems clear that investigations with even larger (and more representative) samples than what we collected in the current research may be necessary to assess some of these predictions about language background.

Relatedly, it is worth emphasizing our theoretical claim that embodiment theory makes predictions about when we should (and should not) see effects of language background on cognitive tasks. That is, it proposes that multilingual speakers should have an advantage in tasks that take advantage of linguistic processes and representations. Conversely, seeing a difference between monolingual and multilingual speakers should be less likely when the task in question does not evoke prior linguistic experiences. Fundamentally, we can only examine the claim—that the use of linguistic materials makes the observation of the multilingual advantage more likely—if we can observe the multilingual advantage in the first place. The fact that we failed to observe such an advantage means that we should be especially cautious about claiming which factors might or might not moderate such an effect. However, we note that our analysis of language dominance, suggested by a reviewer, is perhaps indicative of some effect of linguistic background that our design lacked the sensitivity or the sample to detect.

Finally, and perhaps most importantly, our attempt to differentiate “linguistic” stimuli from “non-linguistic” stimuli suffers from a confound. In all cases, our linguistic materials were presented in English, which is (necessarily) the primary and dominant language for our monolingual speakers, while our multilingual speakers varied more widely in their familiarity with and use of the English language. This may explain why we found an unexpected monolingual advantage in conflict resolution in the Simon task, as the conflict was always expressed with English words. To better understand differences in performance between monolingual and multilingual participants, it may be necessary—as suggested by a thoughtful reviewer—to assess performance in a non-linguistic version of the task.

Indeed, the very embodiment perspective we have advocated suggests that we miss an important avenue toward explanatory power when we classify participants by abstract terms such as “monolingual” or “multilingual,” even when those terms are quantified in relatively continuous ways. Beyond this, we need to think about participants’ goals, their expectations, and how their prior experiences relate to specific tasks. Doing so, we believe, will enable us to make more confident predictions about when prior linguistic experience will transfer to a task, and when it will not. While our current results do not demonstrate this principle as effectively as we hoped, we do believe that these results, in conversation with some of the other topics raised in this special issue, may point toward a useful path forward. Repeatedly, we found that where there was a “bilingual advantage,” it was for stimuli that were less determinedly linguistic (images rather than words; visual digits rather than spoken digits in our span task). In debriefing afterward, however, several participants told us that these “less” linguistic stimuli actually resulted in the participant doing more linguistic processing (trying to self-generate a label). Or, to put it more broadly, a task is linguistic not because of the stimuli in the task, but because of how the participant perceives, represents, and manipulates those stimuli. The embodiment perspective, with its focus on exactly this level of analysis, may provide a productive avenue of defining the cognitive processes associated with language, and thus making predictions about which tasks should show effects of linguistic experience.

## Data availability statement

The original contributions presented in the study are included in the article/Supplementary material, further inquiries can be directed to the corresponding author.

## Ethics statement

The studies involving human participants were reviewed and approved by the Carnegie Mellon University Institutional Review Board. The patients/participants provided their written informed consent to participate in this study. Written informed consent was obtained from the individual(s) for the publication of any potentially identifiable images or data included in this article.

## Author contributions

ET was the primary investigator in the lab where this work occurred, and helped to direct implementation. YK did important conceptual and pragmatic work to bring the task switching methodology into the paradigm. ZY was responsible for the final set of analyses, building upon work by ZL and W-JW. All authors contributed to the article and approved the submitted version.

## Funding

The publication of this work is supported by the Carnegie Mellon University library system.

## Conflict of interest

The authors declare that the research was conducted in the absence of any commercial or financial relationships that could be construed as a potential conflict of interest.

## Publisher’s note

All claims expressed in this article are solely those of the authors and do not necessarily represent those of their affiliated organizations, or those of the publisher, the editors and the reviewers. Any product that may be evaluated in this article, or claim that may be made by its manufacturer, is not guaranteed or endorsed by the publisher.
