# Supplementary material for: Effects of language background on executive function: Transfer across task and modality
Source: Front Psychol. 2023 Jan 5;13:923123. doi: 10.3389/fpsyg.2022.923123 (PMC9849579; doi:10.3389/fpsyg.2022.923123)
Supplement: Supplementary file 2 [file Presentation_1.pdf]

Language Questionnaire

This Language Questionnaire is based on the LEAP-Q (Marian et al., 2007). Notes are italicized.

The goal of this questionnaire is to assess your experience and proficiency in each of the languages you speak. If you are a monolingual speaker, these responses may be repetitive, but we are interested in your answers as well! Please answer the questions as accurately as possible. **If a question that does not apply to you, please respond with "N/A"**

• How many languages do you have an understanding of?
The following options were given as multiple choice; participants were only able to select one option.
    1, 2, 3, 4 or more
(selection becomes "NumLanguages")

You have specified that you have an understanding of _____ language(s).
Press 'Back' if you have entered an incorrect number of languages. Otherwise, press 'Next' to continue.

Language Acquisition

If NumLanguages = 1:
    On the following page, you will answer some questions about your language acquisition.
    Please enter the name of your native language:
    (Text box)

If NumLanguages > 1:
    Please list all the languages you know IN THE ORDER THAT YOU LEARNED THEM. Your native language (the language you learned from your parents), should be FIRST:
        Language 1:
        (Text box)
        Language 2:
        (Text box)
    Appropriate number of text boxes based on NumLanguages. In the example above, NumLanguages = 2.

    The following three questions ask about your CURRENT LANGUAGE HABITS. Please answer accordingly. As a reference point, think about your behavior across an "average" week using the past month as a reference point. All answers should sum to 100%.
    The following questions used a sliding scale from 0 - 100% for each language entered by the participant.

- Please estimate what percentage of the time you CURRENTLY spend SPEAKING each language.
- Please estimate what percentage of the time you CURRENTLY spend LISTENING to each language. Either in conversations or through music, audio, or video recordings.

- Please estimate what percentage of the time you CURRENTLY spend READING in each language (on a weekly basis).

The following 2 questions ask about your CURRENT LANGUAGE PREFERENCES. Please answer according to your AVERAGE preferences, using the past month as a reference point.

- When choosing to read a text available in all your languages, assuming that all versions were translated from some unknown language, which language would you prefer to read in?
  (Text box)
- When choosing a language to speak to a person who is EQUALLY FLUENT in all of your languages, which language would you prefer to speak in?
  (Text box)

Order of Dominance

Please list all the languages you know IN ORDER OF DOMINANCE. Put the language you are MOST COMFORTABLE communicating in under 1, regardless of the order in which you learned it.

Language 1:
(Text box)
Language 2:
(Text box)

Appropriate number of text boxes based on NumLanguages. In the example above, NumLanguages = 2.

Regardless of NumLanguages:

Reminder: Answer "N/A" if a question does not apply to you.
This set of questions were repeated for each language participant reported. Questions 1 - 7 used text box entry. Questions 8 - 10 provided choices 0 through 10.

1. At what age did you begin ACQUIRING ____?
2. At what age did you become FLUENT in ____?
3. At what age did you begin READING in ____?
4. At what age did you become FLUENT in READING in ____?
5. Please list the number of years and months you spent IN A COUNTRY where ____is spoken:
6. Please list the number of years and months you spent WITH A FAMILY where ____is spoken:
7. Please list the number of years and months you spent in a SCHOOL OR WORK ENVIRONMENT where ____is spoken:
8. On a scale of 0 (no skills) to 10 (extremely proficient), please rate your level of proficiency in SPEAKING ____:
9. On a scale of 0 (no skills) to 10 (extremely proficient), please rate your level of proficiency in UNDERSTANDING ____:
10. On a scale of 0 (no skills) to 10 (extremely proficient), please rate your level of proficiency in READING ____:

Language Skills

In the following section, you will read statements about your language skills. If the statement is TRUE FOR YOU, mark 'True'. If the statement is NOT TRUE FOR YOU, mark 'Not True'.
The following set of questions were repeated for each language participant reported. Participants could either select "True" or "Not True".

1. You can understand several words (up to 500) in ______ when they are written (e.g., on signs), or produced in isolation?
2. You can understand ______ word combinations well enough to recognize highly familiar questions, such as "What's your name?" or "Where are you from?".
3. You can understand ______ well enough to follow a basic set of instructions given by a native speaker, such as "How to use a vending machine to buy a ticket" or "Where to find a good deal on shoes"?
4. You understand ______ well enough to follow a casual conversation with a native speaker, such as listening to them tell a story about the town they grew up in and how the environment was different from the town they're in now?
5. You understand ______ enough to follow more in-depth conversations with native speakers, covering a variety of topics?
6. You understand ______ well enough to follow movies and TV shows in this language without subtitles?
7. ______ is either your dominant (strongest) language or you understand this language as well as your strongest language. When listening to someone speaking in this language, you do not have to mentally "translate" the message into a more dominant language. Instead, the meanings of the message are instantly available. You also understand "slang" or regional terms?
8. You can produce several (up to 500) words in ______?
9. You can produce highly familiar questions in ______, such as "What's your name?" or "Where are you from?"
10. You can produce enough basic, familiar, statements to travel in a country that speaks ______. For example, you can order food from a restaurant menu or you could ask how much something costs?
11. You speak ______well enough to have a casual conversation with, and be understood by, a native speaker, such as telling them about your recent travel experiences or something interesting that happened to you earlier that day?
12. You speak ______well enough to have in depth conversations with native speakers covering a variety of topics, such as politics, arts, or science?
13. You speak ______well enough to date someone who only speaks this language, without difficulties?
14. Is there anything else you wish to share?

Demographics Questionnaire

Demographic Information
Please fill out the following demographic questions.

What is your age?
(Text box)

What is your gender?
(Text box)

Which of the following categories describe you?
The following options were check-boxes; participants were able to select multiple options.

- ☐ American Indian or Alaska Native - For example: Navajo Nation; Blackfeet Tribe; Mayan; Aztec; Native Village of Barrow Inupiat Traditional Government; Nome Eskimo Community
- ☐ Black or African American - For example: Jamaican; Haitian; Nigerian; Ethiopian; Somalian
- ☐ Hispanic; Latino; or Spanish Origin - For example: Mexican or Mexican American; Puerto Rican; Cuban; Salvadoran; Dominican; Columbian
- ☐ East Asian - For example: Chinese; Korean; Japanese; Taiwanese; Mongolian
- ☐ Southeast Asian - For example: Vietnamese; Filipino; Indonesian; Thai; Malaysian
- ☐ South Asian - For example: Indian; Afghan; Pakistani; Bangladeshi; Nepalese; Bhutanese; Maldivian; Sri Lankan
- ☐ Middle Eastern or North African - For example: Lebanese; Iranian; Egyptian; Syrian; Qatari; Moroccan; Algerian
- ☐ Native Hawaiian or Other Pacific Islander - For example: Native Hawaiian; Samoan; Chamorro; Tongan; Fijian; Marshallese
- ☐ White - For example: German; Irish; English; Italian; Polish; French

Which Nationality do you most identify with?
The following options were listed as a "dropdown"; participants were only able to select one option.

Afghanistan, Albania, Algeria, American Samoa, Andorra, Angola, Anguilla, Antarctica, Antigua and Barbuda, Argentina, Armenia, Aruba, Australia, Austria, Azerbaijan, Bahamas, Bahrain, Bangladesh, Barbados, Belarus, Belgium, Belize, Benin, Bermuda, Bhutan, Bolivia, Bosnia and Herzegovina, Botswana, Bouvet Island, Brazil, British Indian Ocean Territory, Brunei Darussalam, Bulgaria, Burkina Faso, Burundi, Cambodia, Cameroon, Canada, Cape Verde, Cayman Islands, Central African Republic, Chad, Chile, China, Christmas Island, Cocos (Keeling) Islands, Colombia, Comoros, Congo, Cook Islands, Costa Rica, Croatia (Hrvatska), Cuba, Cyprus, Czech Republic, Denmark, Djibouti, Dominica, Dominican Republic, East Timor, Ecuador, Egypt, El Salvador, Equatorial Guinea, Eritrea, Estonia, Ethiopia, Falkland Islands (Malvinas), Faroe Islands, Fiji, Finland, France, France Metropolitan, French Guiana, French Polynesia, French Southern Territories, Gabon, Gambia, Georgia, Germany, Ghana, Gibraltar, Guernsey,

Greece, Greenland, Grenada, Guadeloupe, Guam, Guatemala, Guinea, Guinea-Bissau, Guyana, Haiti, Heard and Mc Donald Islands, Honduras, Hong Kong, Hungary, Iceland, India, Isle of Man, Indonesia, Iran (Islamic Republic of Iran), Iraq, Ireland, Israel, Italy, Ivory Coast, Jersey, Jamaica, Japan, Jordan, Kazakhstan, Kenya, Kiribati, Korea (Democratic People's Republic of Korea), Korea (Republic of Korea), Kosovo, Kuwait, Kyrgyzstan, Lao People's Democratic Republic, Latvia, Lebanon, Lesotho, Liberia, Libyan Arab Jamahiriya, Liechtenstein, Lithuania, Luxembourg, Macau, Macedonia, Madagascar, Malawi, Malaysia, Maldives, Mali, Malta, Marshall Islands, Martinique, Mauritania, Mauritius, Mayotte, Mexico, Micronesia (Federated States of Micronesia), Moldova (Republic of Moldova), Monaco, Mongolia, Montenegro, Montserrat, Morocco, Mozambique, Myanmar, Namibia, Nauru, Nepal, Netherlands, Netherlands Antilles, New Caledonia, New Zealand, Nicaragua, Niger, Nigeria, Niue, Norfolk Island, Northern Mariana Islands, Norway, Oman, Pakistan, Palau, Palestine, Panama, Papua New Guinea, Paraguay, Peru, Philippines, Pitcairn, Poland, Portugal, Puerto Rico, Qatar, Reunion, Romania, Russian Federation, Rwanda, Saint Kitts and Nevis, Saint Lucia, Saint Vincent and the Grenadines, Samoa, San Marino, Sao Tome and Principe, Saudi Arabia, Senegal, Serbia, Seychelles, Sierra Leone, Singapore, Slovakia, Slovenia, Solomon Islands, Somalia, South Africa, South Georgia South Sandwich Islands, Spain, Sri Lanka, St. Helena, St. Pierre and Miquelon, Sudan, Suriname, Svalbard and Jan Mayen Islands, Swaziland, Sweden, Switzerland, Syrian Arab Republic, Taiwan, Tajikistan, Tanzania (United Republic of Tanzania), Thailand, Togo, Tokelau, Tonga, Trinidad and Tobago, Tunisia, Turkey, Turkmenistan, Turks and Caicos Islands, Tuvalu, Uganda, Ukraine, United Arab Emirates, United Kingdom, United States, United States minor outlying islands, Uruguay, Uzbekistan, Vanuatu, Vatican City State, Venezuela, Vietnam, Virgin Islands (British), Virgin Islands (U.S.), Wallis and Futuna Islands, Western Sahara, Yemen, Yugoslavia, Zaire, Zambia, Zimbabwe

How many pets are in your current household?
The following options were given as multiple choice; participants were only able to select one option.

0, 1, 2, More than 2

From what country are you currently doing this experiment?
(Text box)
